# On the identity and typification of *Solanum brasilianum* Dunal (Solanaceae)

**DOI:** 10.3897/phytokeys.76.11031

**Published:** 2017-01-05

**Authors:** Suelma Ribeiro-Silva, Sandra Knapp, Carolyn E.B. Proença

**Affiliations:** 1 Centro Nacional de Pesquisa e Conservação da Biodiversidade do Cerrado e Caatinga (CECAT), Instituto Chico Mendes; Prédio do Centro de Excelência do Cerrado; Jardim Botânico de Brasília, SMDB Cj 12, Lago Sul, Brasília, Distrito Federal, 70297-400, Brazil; 2 Department of Life Sciences, Natural History Museum, Cromwell Road, London SW7 5BD, United Kingdom; 3 Departamento de Botânica, Instituto de Ciências Biológicas, Universidade de Brasília, C.P. 4457 Brasília, Distrito Federal, 70910-900, Brazil

**Keywords:** Brazil, epitype, Leonard Plukenet, nomenclature, Solanum, William Dampier, William Sherard

## Abstract

*Solanum brasilianum* Dunal was described by Dunal in 1813 with reference only to an illustration in an 18^th^ century work by Leonard Plukenet. The plate is difficult to interpret and no explicitly related specimens were available so the name *Solanum brasilianum* has long been regarded as “unresolved” and has never been used. Material matching the Plukenet plate was discovered in the herbarium of the University of Oxford (OXF) by Stephen Harris during his study of the English privateer William Dampier’s Brazilian collection. The specimen is referable to a common Brazilian *Solanum* that is a member of the Torva clade, *Solanum paniculatum* L., making *Solanum brasilianum* Dunal a heterotypic synonym. We lectotypify *Solanum brasilianum* here, and designate an epitype using the Dampier material from OXF.

## Introduction

Brazil is one of the hotspots of species richness for the mega-diverse genus *Solanum* L. (Solanaceae), with 272 accepted species (Flora do Brasil 2020, http://floradobrasil.jbrj.gov.br/reflora/floradobrasil/FB14716). Much recent work has gone into the resolution of names in the genus (e.g., Knapp et al. 2015), in preparation for the Flora do Brasil 2020 project, and only a few *Solanum* names remain without status in recent updates. Most of these are names attributed to the Italian naturalist Domenico Agostino Vandelli (1735-1816), who worked in Coimbra, Portugal in the mid-18^th^ century, where many new plants from Brazil arrived to Europe (Guimarães 2016; see Solanaceae Source http://www.solanaceaesource.org or Flora do Brasil 2020 http://floradobrasil.jbrj.gov.br/ for these names). Another of these unresolved names is Michel-Félix Dunal’s (1813) *Solanum brasilianum*, whose identity we resolve here.

## *Solanum brasilianum* Dunal

**Figure 1.**
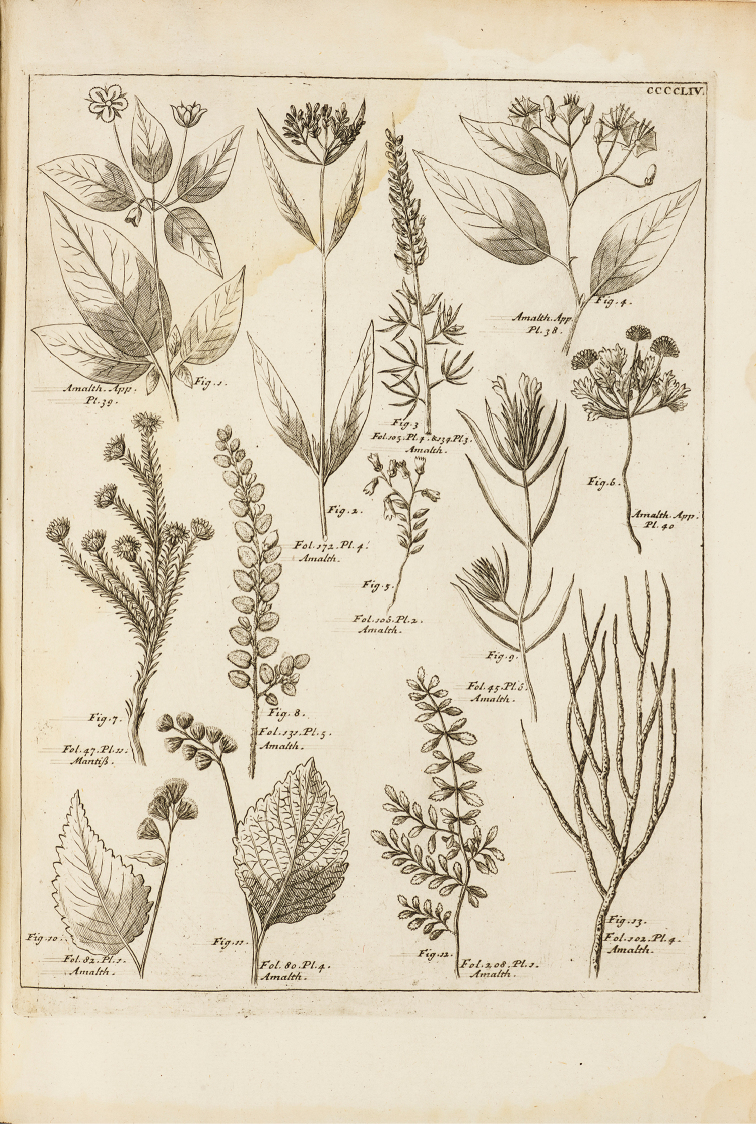
Plukenet, L. 1705. *Amaltheum botanicum* tab. 454, f. 4.

Michel-Félix Dunal described *Solanum brasilianum* citing as his only material a figure (“t. 454, f. 4”) from Plukenet’s (1705) *Amaltheum botanicum* (Dunal 1813: 239) corresponding to the polynomial “Solanum Brasilianum, folio integro mucronato glabro. Papas *Americanui* floribus in summitate caudis.” Plukenet’s polynomial appeared in the Appendix to the *Amaltheum botanicum* (Plukenet 1705) along with other Brazilian and Australian plants based on the collections of William Dampier and Chinese plants sent by Jacob Cunningham (“cum multis aliis in hac appendice recensitis, quae ex Hollandia Nova, atque Brasilia a D. Dampier fibi allatae, necnon ex Insula Cheusan a laudatissimo viron Domino Jacobo Cunningham sunt trasnmissae”: Plukenet 1705: pp. 215). Dunal (1813) extended the polynomial with observations he took directly from the figure – “In figura: folia ovate, suminate, inermia; flores corymbosi; corolla pentagona; antherae divaricatae”. He placed *Solanum brasilianum* amongst his armed species of uncertain status, due to the scarce information available. Plukenet’s (1705) figure is extremely diagrammatic, and has none of the diagnostic features that would enable placement in a species group of *Solanum* (Fig. 1); the polynomial however does allow its placement in *Solanum* by reference to its similarity with potatoes (“Papas *Americani*”).

Otto Sendtner, in *Flora brasiliensis* (Sendtner 1846: 112) also treated *Solanum brasilianum* as a name of uncertain status, and extended Dunal’s (1813) description, still only using the Plukenet illustration as his basis for recognizing the species. He compared it to the Mexican species *Solanum glaucescens* Zucc. (see Clark et al. 2015) based on its curved spines at the leaf bases [“Figura refert ramulum (ex habitu fere *Solanum glaucescentis*) aculeo uno recurvo ad folii basin, foliis petiolatis ovato-lanceolatis acuminatis basi acutiusculis 6-7 nervilis solitariis; inflorescentiam corymbiformem, subapicalem 8-floram, repetito-dichotomam, pedunculo comuni breviori quam secundarii; pedicellos graciles; alabastra oblonga obtusa; calycem 5-fidum vel partitum? laciniis acutiusculis, corollam magnam 5-angularem, antheras longas, angustas, lineares, corollam aequantes.”]. He misinterpreted the small axillary leaf shoots for curved spines; the plate is of an unarmed plant as Dunal later recorded in his treatment for the *Prodromus* (Dunal 1852: 372), placing it among unarmed species, but still of uncertain status.

### Dampier’s specimen

**Figure 2.**
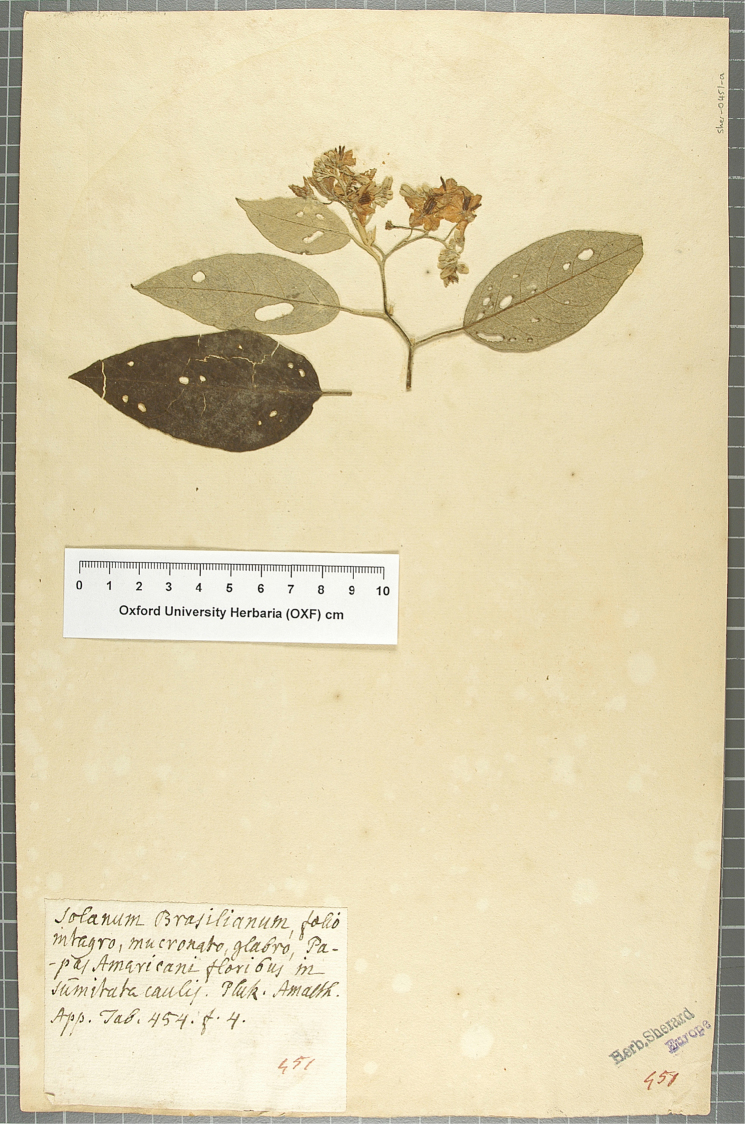
Brazil. Bahia: Salvador, April-May 1699, *W. Dampier* “herb. Dampier no. 451” (epitype, designated here: OXF! [Sher-0451-a]).

William Dampier was an English privateer and navigator who circumnavigated the globe three times between 1686 and 1710 (Preston and Preston 2005). He was a keen observer of nature and during his travels HMS Roebuck Dampier collected a handful of plant specimens from Australia and Brazil. These he gave to John Woodward, a professor at Gresham College, who later gave them to the botanist William Sherard, who in turn bequeathed them to Oxford University when he died in 1728 (Harris et al. 2016). They are now kept in the Sherardian Herbarium at OXF. During a study of these historical collections, Stephen Harris (OXF) found a specimen collected by William Dampier in Salvador, Bahia, during his time in Brazil in April-May 1699 (Harris et al. 2016). The sheet is a single specimen with consisting of a small branch with three attached leaves and a single leaf not attached the branch, but clearly belonging to it (Fig. 2). The specimen has a label with the polynomial taken from Plukenet (1705) “Solanum Brasilianum, folio integro macronato glabro, Papas Americani floribus in summutate caulis. Pluk. Amalth. App. Tab. 454, f. 4” in Sherard’s hand. The specimen is a good match for the illustration in Plukenet (1705) and is likely to have been the specimen from which that plate was made. Plukenet was based in London at the time the *Amaltheum botanicum* was begin prepared (Jarvis 2007), and he was shown Dampier’s specimens by his “learned friend [*Amicissimus & eruditis Vir*]” John Woodward (Plukenet 1705: 215).

The branch shown in plate 454, fig. 4 of Plukenet’s *Amaltheum botanicum* (1705) is completely unarmed, has leaves with entire margins that are markedly discolourous, and are adaxially glabrescent and abaxially densely stellate-tomentose. The small axillary buds above the lower leaves are likely the elements misinterpreted by Sendtner (1846) as spines at the leaf bases (see Fig. 2). The open flowers have anthers like those of *Solanum paniculatum* (and other members of the Torva clade sensu Stern et al. 2011) that characteristically spread when dry. These morphological characteristics clearly show that the specimen belongs to the taxon currently recognized as *Solanum paniculatum* L.. *Solanum paniculatum* was described by Linnaeus in his second edition of *Species Plantarum* (1762: 267) based on an illustration “Jurepeba” from Willem Piso’s *De Indiae utrisque re naturali et medici libri 14* (Piso 1658; see Knapp and Jarvis 1991). *Solanum paniculatum* is a common small tree or shrub occurring in all phytogeographical areas and regions of Brazil (Flora do Brasil 2020 http://floradobrasil.jbrj.gov.br/jabot/floradobrasil/FB127325) and northeastern Argentina and eastern Paraguay (http://www.solanaceaesource.org). It is also extremely variable morphologically, with leaf shapes in particular varying from deeply lobed to entire, even on an individual plant (see Fig. 16 in Knapp and Jarvis 1991). The Dampier specimen has four entire leaves, with glabrescent upper leaf surfaces beearing sparse stellate trichomes, and their shape is almost identical to distal portions of stems from a modern specimen (e.g. *Medeiros Neto 27* at VIES) of *Solanum paniculatum* from Bahia and, like Dampier’s specimen, this very similar plant was collected with flowers in April.

Since Dunal (1813) did not have access to the Dampier specimen when he described *Solanum brasilianum* the Plukenet image (Fig. 1, upper right hand illustration, “Tab. 454, f. 4”) is the only original material (McNeill et al. 2012) and we select it here as the lectotype. Because the illustration is so diagrammatic, and the Dampier specimen is clearly that from which it was prepared, we select the specimen “Herb. Dampier n. 451”, collected by Dampier, as the epitype. Thus the discovery of this long-neglected specimen of *Solanum brasilianum* and its examination have allowed us to elucidate its true identity, and we here recognize *Solanum brasilianum* as a heterotypic synonym of *Solanum paniculatum*.

## Taxonomic treatment

### Solanum brasilianum

Taxon classificationPlantaeSolanalesSolanaceae

Dunal, Hist. Nat. Solanum 239. 1813.

#### Type.

Brazil. Sin. loc., no collector cited (lectotype, designated here: Plukenet, L. 1705. *Amaltheum botanicum* tab. 454, f. 4; epitype, designated here: Brazil. Bahia: Salvador, April-May 1699, *W. Dampier* “herb. Dampier no. 451”[OXF! (Sher-0451-a)]).

#### Current accepted name.

*Solanum paniculatum* L.

## Supplementary Material

XML Treatment for Solanum brasilianum

## References

[B1] Clark JL Nee M Knapp S (2015) A Revision of Solanum Section Aculeigerum (the *Solanum wendlandii* Group, Solanaceae). Systematic Botany 40(4): 1102-1136. http://dx.doi.org/10.1600/036364415X690148

[B2] Dunal MF (1813) Histoire naturelle, médicale et économique des *Solanum* et des generes qui ont été confondus avec eux. Chez Renaud Libraire, Montpellier, 248 pp.

[B3] Dunal MF (1852) Solanaceae. Prodromus systematis naturalis regni vegetabilis 13(1): 1-690.

[B4] Flora do Brasil 2020 em construção (2016) Jardim Botânico do Rio de Janeiro http://floradobrasil.jbrj.gov.br/reflora/floradobrasil/FB14716 [accessed 04.06.2016]

[B5] Guimarães J (2016) Vandelli, Domenico, 1735−1816. http://www.uc.pt/bib_dep_botanica/biblioteca_digital_botanica/autores_e_personalidades/vandelli [accessed 4.06.2016]

[B6] Harris S Serena K Proença C (2016) William Dampier’s Brazilian botanical observations in 1699. Journal of the History of Collections Advance. https://doi.org/10.1093/jhc/fhw023 [access published July 1, 2016]

[B7] Jarvis CE (2007) Order out of chaos: Linnaean plant names and their types. Linnean Society of London and Natural History Museum, London, 1016 pp.

[B8] Knapp S Jarvis CE (1991 [1990]) The typification of the names of New World species of *Solanum* described by Linnaeus. Botanical Journal of the Linnean Society 104: 325-367. https://doi.org/10.1111/j.1095-8339.1990.tb02227.x

[B9] Knapp S Barboza GE Romero MV Vignoli-Silva M Giacomin LL Stehmann JR (2015) Identification and lectotypification of the Solanaceae from Vellozo’s *Flora Fluminensis*. Taxon 64(4): 822-836. https://doi.org/10.12705/644.14

[B10] Linnaeus C (1762) Species Plantarum (2 edn). Laurentius Salvius, Stockholm, 267 pp.

[B11] McNeill J Barrie FR Buck WR Demoulin V Greuter W Hawksworth DL Herendeen PS Knapp S Marhold K Prado J Prud’homme van Reine WF Smith GF Wiersema JH Turland NJ (2012) International Code of Nomenclature for algae, fungi, and plants (Melbourne Code). Regnum Vegetabile 154. Koelz Scientific Books, Königstein, 240 pp.

[B12] Piso W (1658) De India utriusque re naturali et medici libri 14. Amstelaedami: apud Ludovicum et Danielem Elzevirios, 625 pp.

[B13] Plukenet L (1705) *Amaltheum botanicum*. Pubished by the author, London, 214 pp.

[B14] Preston D Preston M (2005) A Pirate of Exquisite Mind. Explorer, Naturalist, and Buccaneer: the Life of William Dampier. Walker & Company, New York, 372 pp.

[B15] Sendtner O (1846) Solanaceae. In: Martius CFP (Ed.) Flora Brasiliensis. Volume 10, 9–113.

[B16] Stern S Agra MF Bohs L (2011) Molecular delimitation of clades within New World species of the ”spiny solanums” (Solanum subgenus Leptostemonum). Taxon 60: 1429-1441. http://www.ingentaconnect.com/contentone/iapt/tax/2011/00000060/00000005/art00018 10.1002/tax.605018 PMC7169808 32327851

